# Beyond Ketosis: Dietary Therapies and the Microbiota–Gut–Brain Axis in Epilepsy

**DOI:** 10.3390/nu18132151

**Published:** 2026-07-02

**Authors:** Valentina Biagioli, Mariarosaria Matera, Ilaria Imola, Federica Mela, Damiano Lemmi, Alberto Verrotti, Pasquale Striano

**Affiliations:** 1 Department of Neuroscience, Rehabilitation, Ophthalmology, Genetics, Maternal and Child Health, University of Genoa, 16126 Genoa, Italy; pasqualestriano@gaslini.org; 2 Department of Pediatric Emergencies, Misericordia Hospital, 58100 Grosseto, Italy; jajamatera74@gmail.com; 3 Child Neuropsychiatry Unit, Department of Mental Health, Physical and Preventive Medicine, University of Campania “Luigi Vanvitelli”, 81100 Caserta, Italy; imola.ilaria03@gmail.com; 4 Department of Neurosciences, Rehabilitation, Ophthalmology, Genetics and Maternal and Child Health (DINOGMI), University of Genoa, 16126 Genoa, Italy; melafederica97@gmail.com (F.M.); damianolemmi97@gmail.com (D.L.); 5 Department of Paediatrics, University of Perugia, 06123 Perugia, Italy; alberto.verrottidipianella@unipg.it; 6 Pediatric Neurology and Muscular Diseases Unit, Scientific Institute for Research, Hospitalization and Health Care (IRCCS) Istituto Giannina Gaslini, 16147 Genoa, Italy

**Keywords:** epilepsy, microbiota–gut–brain axis, ketogenic diet, modified Atkins diet, low glycemic index treatment, neuroinflammation

## Abstract

**Background:** Epilepsy is a complex neurological disorder in which growing evidence supports a significant role for the microbiota–gut–brain axis (MGBA) in modulating neuroinflammation, neuronal excitability, and treatment responsiveness. Beyond their traditional role in inducing ketosis, dietary therapies may influence epilepsy by modulating gut microbial ecology, intestinal barrier integrity, immune signaling, and microbiota-derived metabolites. **Methods:** This narrative review critically examines current clinical and experimental evidence regarding the relationship between epilepsy, gut microbiota, and dietary interventions. Particular attention was given to ketogenic dietary therapies, the Modified Atkins Diet (MAD), low-glycemic-index treatment (LGIT), Mediterranean dietary patterns, restrictive diets, and microbiota-targeted supplementation, including probiotics, prebiotics, and postbiotics. **Results:** Available evidence suggests that patients with epilepsy exhibit alterations in gut microbial composition associated with impaired short-chain fatty acid production, intestinal inflammation, and altered neuroimmune regulation. Ketogenic and microbiota-supportive dietary approaches may modulate these pathways beyond ketosis alone, potentially contributing to seizure reduction through integrated metabolic, inflammatory, and microbial mechanisms. Emerging evidence also supports a role for probiotics, prebiotics, and postbiotics in modulating gut–brain communication and neuroinflammatory signaling, although current clinical data remain limited. **Conclusions:** Dietary therapies in epilepsy should no longer be viewed exclusively as metabolic interventions aimed at inducing ketosis, but rather as potential modulators of the microbiota–gut–brain axis and neuroimmune homeostasis. While further mechanistic and clinical studies are needed, microbiota-targeted nutritional approaches may represent valuable complementary strategies to be integrated alongside conventional antiseizure therapies within more personalized models of epilepsy management.

## 1. Introduction

Epilepsy is one of the most common neurological conditions worldwide, affecting over 50 million individuals, with an estimated annual incidence of approximately 6 cases per 10,000 people and a recurrence rate exceeding 80% ten years after diagnosis [1].

Despite significant advances in pharmacological treatment in recent decades, approximately 30% of patients continue to experience seizures refractory to available antiepileptic drugs [2]. This persistent proportion of drug-resistant epilepsy highlights a significant unmet clinical need and suggests that current therapeutic strategies are unable to address the biological mechanisms underlying epileptogenesis adequately. In addition to the long-term persistence of seizures, the disease is frequently associated with impaired quality of life, social stigma, neurological and psychiatric comorbidities, and a considerable economic burden related to healthcare costs. These factors contribute significantly to the deterioration of patients’ quality of life, with a particularly marked impact in resource-limited settings [3].

Conventional antiseizure medications were primarily developed to target neuronal hyperexcitability within the central nervous system. Growing evidence now highlights that neuronal excitability and seizure susceptibility are also influenced by systemic metabolic, immune, and microbial signals originating from the gut microbiota. This axis, called the microbiota–gut–brain axis (MGBA), has emerged as a key bidirectional communication network capable of modulating neuronal excitability, seizure susceptibility, and response to pharmacological treatments [4,5,6].

Through neural, endocrine, immune, and metabolic pathways, the gut microbiota may influence neuroinflammation, blood–brain barrier integrity, neurotransmitter production, and brain homeostasis.

Growing evidence suggests that dysfunction of the MGBA may contribute to chronic inflammatory, metabolic, neurodevelopmental, and neurodegenerative disorders [7,8]. Growing studies have also highlighted a close connection between epilepsy and the gut. Significant differences in the gut microbial composition have been observed between healthy subjects and those with epilepsy. Although a definitive epilepsy-associated microbial signature has not yet been identified, the current findings support the hypothesis that gut dysbiosis may contribute to epileptogenesis and treatment responsiveness.

In parallel, increasing attention has been devoted to dietary therapies for epilepsy not only as metabolic interventions capable of inducing ketosis, but also as potential modulators of the intestinal ecosystem, neuroimmune signaling, and microbiota-derived metabolic pathways. In this context, this review aims to critically examine current clinical and experimental evidence regarding the role of the MGBA in epilepsy, with particular attention to how dietary interventions may influence gut microbiota composition, neuroinflammation, and seizure susceptibility. Furthermore, we discuss the potential advantages and limitations of microbiota-targeted nutritional strategies as complementary approaches in epilepsy management.

## 2. Materials and Methods

A narrative literature search was conducted using the PubMed and Scopus databases to identify publications investigating the relationship between epilepsy, the microbiota–gut–brain axis (MGBA), and dietary interventions. The search included articles published from January 2000 to May 2026 and was performed using combinations of the following keywords: “epilepsy”, “drug-resistant epilepsy”, “gut microbiota”, “microbiota–gut–brain axis”, “ketogenic diet”, “modified Atkins diet”, “low glycemic index treatment”, “Mediterranean diet”, “dietary therapy”, “neuroinflammation”, “probiotics”, “prebiotics”, and “postbiotics”. Boolean operators (AND, OR) were used to optimize the search, and additional relevant publications were identified through manual screening of the reference lists of selected articles.

The literature considered for this review included original research articles, clinical trials, observational studies, systematic reviews, meta-analyses, and experimental animal studies published in English. Publications were selected based on their scientific relevance and their contribution to the understanding of the interactions among epilepsy, gut microbiota, and dietary interventions.

The selected literature was organized into thematic sections addressing gut microbial alterations in epilepsy, intestinal inflammation and neuroinflammation, dietary interventions, and microbiota-targeted strategies, including probiotics, prebiotics, and postbiotics. The evidence was synthesized narratively to provide a comprehensive overview of current knowledge, discuss emerging mechanistic insights, and identify future research directions. Given the narrative nature of this review, the literature search was intended to identify the most relevant evidence rather than to perform a formal systematic review with predefined study selection procedures.

## 3. Integrating Gut Microbiota into Epilepsy Management

### 3.1. Limitations of the Exclusively Pharmacological Approach

Anti-seizure medications (ASMs) remain the cornerstone of epilepsy treatment; however, approximately 30% of patients continue to experience drug-resistant epilepsy (DRE), highlighting the limitations of an exclusively pharmacological approach. Real-world evidence has shown that therapeutic escalation beyond first-line regimens is frequently associated with increasing polytherapy, limited seizure control, poor tolerability, and a substantial burden of adverse events and healthcare resource utilization [9]. These findings emphasize the need for complementary therapeutic strategies capable of targeting the metabolic, inflammatory, immune, and microbial mechanisms increasingly implicated in epileptogenesis and treatment responsiveness.

### 3.2. Gut Microbial Alterations in Epilepsy

Among the emerging mechanisms of interest, the MGBA has received growing attention as a bidirectional communication network involved in neuronal excitability, immune signaling, and metabolic homeostasis. Increasing evidence suggests that patients with epilepsy exhibit distinct gut microbial profiles compared with healthy controls, although findings remain partially heterogeneous across studies.

Zhou et al. demonstrated significant alterations in gut microbial composition in children with newly diagnosed focal epilepsy, characterized by increased abundance of *Actinobacteria* and reduced representation of short-chain fatty acid (SCFA)-producing genera such as *Faecalibacterium* and *Anaerostipes*. Interestingly, some of these alterations partially shifted after oxcarbazepine treatment, suggesting a bidirectional interaction between ASMs and gut microbial composition [10].

Similarly, Riva et al. identified a distinct gut microbiota signature in children with genetic epilepsy, demonstrating differences between medication-resistant and medication-sensitive patients, particularly involving butyrate-producing taxa such as the *Eubacterium siraeum* group. Notably, these findings suggest that specific microbial profiles may serve as potential biomarkers of treatment responsiveness and antiseizure medication resistance, supporting the emerging role of the gut microbiota in precision medicine approaches to epilepsy [11]. Additional studies further supported the presence of recurrent patterns of gut microbial alterations, characterized by enrichment of taxa associated with microbial imbalance and altered mucosal metabolism, including *Akkermansia* and *Proteobacteria*, together with depletion of SCFA-producing bacteria [12,13].

Consistent findings were also reported by Lindefeldt et al. [14] and Lee et al. [15], who observed reduced microbial richness and significant alterations involving Actinobacteria and Bacteroidetes in children with refractory epilepsy. Collectively, these findings suggest that epilepsy is frequently associated with recurrent patterns of gut dysbiosis, although the specific microbial signatures reported across studies remain heterogeneous. While a reduced abundance of SCFA-producing taxa, including *Faecalibacterium* and *Anaerostipes*, has been reported in several cohorts, these findings are only partially reproducible. Variability in patient characteristics, epilepsy syndromes, age, dietary habits, sequencing methodologies, and bioinformatic pipelines likely contributes to the observed heterogeneity. In addition, the potential influence of antiseizure medications (ASMs) represents an important confounding factor, as these drugs may themselves alter gut microbial composition. Consequently, it remains challenging to distinguish microbial signatures associated with epilepsy from those related to pharmacological treatment, highlighting the need for larger, well-controlled longitudinal studies.

### 3.3. From Dysbiosis to Mechanistic Evidence

Although current human studies mainly demonstrate associative rather than causal relationships, experimental evidence has strengthened the biological plausibility of microbiota involvement in epileptogenesis and seizure modulation. In particular, Francesca Mengoni et al. demonstrated that fecal microbiota transplantation from epileptic animals into healthy recipient mice increased susceptibility to status epilepticus after a subclinical dose of pilocarpine [16]. These findings provide compelling experimental evidence that gut microbiota may directly influence neuronal excitability and seizure susceptibility through the microbiota–gut–brain axis signaling pathways.

Overall, growing clinical and experimental evidence supports the concept that the gut microbiota may represent not only a biomarker of disease activity, but also a potential mechanistic contributor to neuroinflammation, seizure susceptibility, and treatment responsiveness in epilepsy. These observations support the emerging rationale for microbiota-targeted interventions as complementary strategies in epilepsy management.

## 4. Intestinal Inflammation and Neuroinflammation in Epileptogenesis

### 4.1. Barrier Dysfunction and the Microbiota–Gut–Brain Axis

The gut microbiota and the brain communicate through interconnected neural, immune, endocrine, and metabolic pathways, collectively known as the MGBA. Through microbial metabolites, immune signaling, and vagal pathways, the gut microbiota contributes to the regulation of intestinal and blood–brain barrier integrity, neuroimmune homeostasis, and neuronal excitability [17]. Experimental studies in germ-free mice have demonstrated increased permeability of the blood–brain barrier together with altered tight junction expression, whereas colonization with normal microbiota restored barrier integrity [18,19]. These findings support the concept that microbial dysbiosis may promote systemic inflammation and neuroinflammatory signaling through disruption of epithelial and vascular barriers.

### 4.2. Peripheral Inflammation and Seizure Susceptibility

Neuroinflammation is increasingly recognized as a central contributor to epileptogenesis and seizure generation. Inflammatory mediators may enhance neuronal hyperexcitability, while seizures themselves can further activate inflammatory pathways within the central nervous system [20,21]. Experimental models have shown that systemic administration of lipopolysaccharide (LPS), a potent inducer of peripheral inflammation, can reduce the seizure threshold and increase seizure susceptibility [22]. Similarly, chronic inflammatory disorders, including inflammatory bowel disease, systemic lupus erythematosus, and Behçet’s disease, have been associated with increased epilepsy risk. Collectively, these findings support the concept that peripheral inflammation and systemic immune activation may contribute to epileptogenesis through amplification of neuroinflammatory pathways and disruption of neuroimmune homeostasis.

### 4.3. Intestinal Inflammation as a Therapeutic Target

Further evidence linking intestinal inflammation to epilepsy derives from experimental studies demonstrating that colitis may increase seizure susceptibility through systemic inflammatory signaling [23]. Interestingly, sodium butyrate showed anticonvulsant effects under inflammatory conditions, supporting the hypothesis that modulation of intestinal inflammation and microbiota-derived metabolites may represent promising complementary therapeutic strategies in epilepsy.

Overall, these findings suggest that intestinal dysbiosis and peripheral inflammation may contribute to epileptogenesis through disruption of barrier integrity, systemic immune activation, and neuroinflammatory signaling. However, most of the available evidence supporting these mechanisms derives from experimental animal studies, and their clinical relevance in humans remains to be fully established. Therefore, microbiota-targeted interventions should currently be regarded as promising investigational strategies rather than established therapeutic approaches, warranting further mechanistic and clinical studies.

## 5. Dietary Therapies Beyond Ketosis: Modulating the Microbiota–Gut–Brain Axis in Epilepsy

### 5.1. Low Glycemic Index Diet

Among dietary approaches investigated in epilepsy, low glycemic index therapies may represent an intermediate strategy capable of combining metabolic stabilization with a less restrictive nutritional profile than classical ketogenic therapies. Whether these characteristics translate into a more favorable effect on gut microbiota composition remains largely hypothetical and has not yet been demonstrated consistently in clinical studies.

A low glycemic index diet (LGID) is defined as a regimen in which meal planning is based on how foods influence blood glucose levels. LGID has been investigated in several metabolic conditions, including diabetes, hypercholesterolemia, and weight management [24]. It has also proven to be of great interest in the management of drug-resistant epilepsy, as it is much easier to administer than the ketogenic diet KD and has shown encouraging results in terms of clinical improvement. A randomized controlled trial published in Jama Pediatrics by Vishal Sondhi et al. [25] on 170 children with drug-resistant epilepsy compared the ketogenic diet (KD), the Modified Atkins Diet (MAD), and Low Glycemic Index Therapy (LGIT) over 24 weeks. The median reduction in seizure frequency was 66% in the KD group and 54% in the LGIT group, with no statistically significant differences between the groups. This finding should be interpreted with caution, as this study may not have been adequately powered to detect modest differences in efficacy between the dietary interventions. In addition, factors such as variability in epilepsy syndromes, baseline clinical characteristics, and dietary adherence may have influenced the observed outcomes. Further comparative studies with larger, well-characterized cohorts are needed to clarify whether one dietary approach offers superior antiseizure efficacy. Of particular clinical importance, LGIT showed a more favorable safety profile, with a lower incidence of adverse events than KD and MAD. Overall, LGIT emerges as an effective and better-tolerated therapeutic option, supporting an individualized approach in the choice of dietary therapies for drug-resistant epilepsy. However, most available studies have been conducted in pediatric DRE cohorts, and whether similar benefits extend to adult patients remains insufficiently explored.

Furthermore, a recent systematic review with meta-analysis [26], conducted according to the Preferred Reporting Items for Systematic Reviews and Meta-Analyses (PRISMA) guidelines, evaluated the efficacy of LGID in reducing seizure frequency in pediatric age, including 13 studies published between 2005 and 2021. The duration of the interventions ranged from 6 to 58 weeks. The pooled analysis showed that 34% of patients achieved a seizure reduction of ≥50%, while a reduction of >90% was observed in 19% of cases. The results also suggest greater efficacy of LGID in shorter intervention periods (<12 weeks). A recent review of randomized controlled trials compared the MAD and LGIT in children with drug-resistant epilepsy [27]. It included three randomized controlled trials (RCTs) with a total of 265 participants. Pooled analyses showed comparable efficacy between the two diets in terms of seizure frequency reduction, percentage of seizure-free patients, and response rates of ≥50% or ≥90%. However, the MAD was associated with a higher incidence of adverse events, while the LGIT showed numerically higher adherence rates. Overall, these results support LGIT as an effective and potentially better-tolerated alternative to the MAD in children with drug-resistant epilepsy. A preclinical study conducted in a mouse model of generalized epilepsy (GABAAγ2 R43Q mutation) evaluated the effect of LGID on seizure susceptibility [28]. After three weeks of dietary intervention, mice fed a low-glycemic index diet showed an approximately 35% reduction in spike-wave discharges compared to the high-glycemic index group, while no differences emerged between the high-glycemic index and standard diets. Tolerability was comparable between the groups. However, under conditions of induced hypoglycemia, the protective effect of the low-glycemic index diet was abolished. These results suggest that the anticonvulsant action of LGID may be mediated, at least in part, by stabilizing blood glucose levels, providing a biological rationale to support the clinical observations.

Compared with more restrictive ketogenic approaches, LGIT may exert part of its therapeutic effects through improved glycemic stability. Its potential influence on gut microbiota composition has been hypothesized on the basis of its less restrictive dietary profile and greater fiber intake compared with classical ketogenic therapies; however, direct evidence supporting microbiota-mediated effects remains limited. Further clinical studies integrating microbiome analyses are needed to clarify whether modulation of the gut microbiota contributes to the therapeutic effects of LGIT. Nevertheless, evidence regarding its direct impact on microbiota composition and microbiota-mediated modulation of epileptic activity remains limited and is still mainly derived from experimental models. In light of growing evidence on the importance of the MGBA in modulating neuronal function, further clinical studies exploring the contribution of the microbiota appear necessary as a possible mediator of clinical outcomes in drug-resistant epilepsy, particularly in response to the introduction of specific dietary regimens.

### 5.2. Modified Atkins Diet (MAD)

#### 5.2.1. Dietary Characteristics and Ketogenic Principles

The MAD consists of a less restrictive ketogenic dietary approach characterized by high fat intake and reduced carbohydrate consumption, aimed at inducing nutritional ketosis while improving tolerability and long-term adherence compared with the classical ketogenic diet (KD) [29]. It is an alternative to the classic ketogenic diet because it is more flexible and palatable, leading to fewer dropouts. Moreover, it is called “modified” to distinguish it from the traditional Atkins Diet, which was originally designed for weight loss [30].

#### 5.2.2. Neuroprotective and Immunometabolic Mechanisms

Beyond ketone body production alone, MAD aims to induce and maintain a stable state of nutritional ketosis while also influencing interconnected metabolic, neuroimmune, and microbiota-related pathways, characterized by increased systemic production and cerebral utilization of ketone bodies.

Ketosis is thought to exert anticonvulsant and neurostabilizing effects through multiple, potentially complementary mechanisms, although the relative contribution of each pathway remains incompletely understood. By shifting neuronal energy metabolism from glucose to ketone bodies, it provides a more stable and efficient source of ATP, thereby reducing metabolic fluctuations that may facilitate neuronal hyperexcitability. Ketosis has been proposed to enhance inhibitory neurotransmission by increasing γ-aminobutyric acid (GABA) availability and modifying the glutamate/GABA balance. Activation of ATP-sensitive potassium (KATP) channels has also been proposed as a mechanism contributing to neuronal hyperpolarization and reduced excitability. In addition, ketosis improves mitochondrial function and reduces oxidative stress, both of which are recognized contributors to neuronal excitability. Excitatory signaling is further modulated by indirect inhibition of N-methyl-D-aspartate (NMDA) receptor activity and limitation of intracellular Ca^2+^ influx.

Ketone bodies, particularly β-hydroxybutyrate, have also been suggested to exert epigenetic and anti-inflammatory effects, including inhibition of histone deacetylases (HDACs), although the clinical relevance of these mechanisms in epilepsy remains to be fully established [31].

Emerging evidence also suggests that some of these metabolic and anti-inflammatory effects may be partially mediated through modulation of the gut microbiota and microbiota-derived metabolites. Ketone bodies may influence gut microbial ecology by promoting taxa associated with anti-inflammatory and neuroprotective metabolic pathways, further reinforcing the bidirectional relationship between ketosis and the MGBA. Overall, these mechanisms should be regarded as complementary and potentially interacting rather than mutually exclusive. Although preclinical and experimental evidence support their biological plausibility, the relative contribution of each pathway to the antiseizure effects of ketogenic therapies in humans remains uncertain and requires further investigation.

#### 5.2.3. Clinical Evidence in Drug-Resistant Epilepsy

Recent studies confirm that MAD is an effective treatment for drug-resistant epilepsy (DRE). Therefore, most available evidence derives from pediatric populations, particularly children with developmental and epileptic encephalopathies and refractory focal epilepsies, whereas adult data remain comparatively limited.

DRE is defined as the failure of two tolerated, appropriately chosen, and used antiepileptic drug schedules, either in monotherapies or drug combinations, to achieve sustained seizure freedom [32].

Meena et al. demonstrated significant seizure reduction in children treated with MAD compared with topiramate, with both interventions showing acceptable tolerability profiles [33].

Preliminary evidence in adult patients also suggests potential benefits of MAD in psychogenic non-epileptic seizures (PNESs), particularly regarding anxiety, depression, and quality of life measures [34,35]; additional studies also reported improvements in quality of life, emotional well-being, and behavioral outcomes in patients treated with MAD [36].

Other studies explored the difference between treatments with MAD and classic Ketogenic Diet (KD). Comparable efficacy between MAD and KD has been consistently reported, while MAD generally demonstrated superior tolerability, adherence, and fewer adverse events [37,38]; similar findings were also observed in additional comparative studies evaluating MAD and KD in pediatric DRE populations [39].

#### 5.2.4. Microbiota-Targeted Perspectives

Collectively, these findings suggest that MAD may represent a more sustainable alternative to classical ketogenic therapies, combining antiseizure efficacy with improved tolerability, adherence, and quality of life. Beyond metabolic adaptation alone, increasing evidence suggests that ketosis-induced modulation of neuroinflammatory pathways and gut microbial ecology may contribute to its therapeutic effects, further supporting the role of KDTs within the microbiota–gut–brain axis framework.

#### 5.2.5. Microbiota Modulation Induced by MAD

The efficacy of MAD is also related to the changes that it can make in microbiota composition; for example, this type of diet reduces *Actinobacteria* and decreases *Proteobacteria* relative abundance; the lack of *Proteobacteria* is related to decreased serum total oxidant status, thus a diminution of inflammation, and in particular neuroinflammation, which is related to epilepsy [40]. MAD has also been associated with increased production of SCFAs by enriching microbial taxa involved in their synthesis, potentially contributing to intestinal homeostasis and neuroimmune regulation. Unlike the Western diet, MAD has been associated with changes in microbial metabolic pathways involved in the biosynthesis of neurotransmitter precursors, including phenylalanine, tyrosine, and tryptophan. Although these findings suggest a potential influence on microbiota–gut–brain communication, their clinical relevance for seizure control remains uncertain, and no direct evidence currently links these microbial metabolic changes to improved epilepsy outcomes. Moreover, MAD induces directional shifts in the metabolic flux of additional neurotransmitter precursors, including L-tryptophan, in bacterial taxa such as *Bacillus licheniformis* and *Dorea formicigenerans*, suggesting a potential influence on serotonergic and dopaminergic signaling within the MGBA [41]. The impact of MAD on gut microbiota composition has also been investigated in the context of antibiotic-associated *Clostridioides difficile* infection. Evidence suggests that MAD may increase susceptibility to *C. difficile* overgrowth, potentially due to the depletion of taxa that normally inhibit its proliferation and to an increased luminal availability of amino acids that support its expansion [42]. Furthermore, a study by Yu et al., evaluated the effects of MAD in participants, some of them achieving clinically significant weight loss. Participants who lost at least 10% of their body weight exhibited increased bacterial and fungal gut diversity. These microbial shifts involved taxa belonging to Firmicutes and Bacteroidetes, two phyla widely implicated in metabolic and neuropsychiatric disorders. Given the established involvement of these microbial *phyla* in multiple neuropsychiatric conditions, these findings underscore the potential role of MAD in modulating the gut–brain axis [43].

Collectively, these findings suggest that MAD may influence gut microbial composition and metabolic pathways beyond ketosis alone. However, whether these microbiota-related changes contribute directly to seizure reduction remains to be established, and the available evidence does not yet allow a causal relationship between microbial remodeling and clinical outcomes to be inferred.

#### 5.2.6. Limitations of the Modified Atkins Diet (MAD)

Even if it is less restrictive than the classic Ketogenic Diet, nutritional deficiencies may still emerge during long-term adherence to MAD, particularly involving calcium and vitamin D intake in pediatric patients [44]. Moreover, the deficiency of calcium, vitamin D, and other micronutrients essential for bone health increases the risk of bone demineralization and osteopenia, which were found to be common in children following MAD. Other potential long-term consequences of these deficiencies are weakened immunity, metabolic disturbances, and gastrointestinal issues such as constipation, dehydration, and electrolyte imbalances [45]. Findings highlight the importance of long-term nutritional monitoring, preservation of dietary quality and diversity, and routine assessment of micronutrient status in patients undergoing ketogenic dietary therapies.

### 5.3. Ketogenic Therapies Beyond Ketosis: The Role of Gut Microbiota

#### 5.3.1. Ketogenic Therapies as Metabolic and Neuroimmune Interventions

Ketogenic dietary therapies (KDTs) have been employed to treat epilepsy since the 1920s–1930s, although their use declined with the advent of new anti-seizure medications. Interest in these approaches resurged in the 1990s following encouraging clinical evidence, and KDTs are now recognized worldwide as effective therapeutic alternatives for patients with refractory epilepsy who are poor surgical candidates. Their strongest evidence base currently exists in pediatric drug-resistant epilepsies, including developmental and epileptic encephalopathies such as Dravet syndrome, Lennox–Gastaut syndrome, and glucose transporter type 1 deficiency syndrome glucose transporter type 1 deficiency syndrome (GLUT1-DS), whereas evidence in adults remains comparatively limited, although a growing number of studies support their use in selected adult populations [46].

The term “KDTs” currently encompasses multiple dietary protocols differing in ketogenic ratios and food composition, including the classical ketogenic diet (cKD), medium-chain triglyceride ketogenic diet (MCT-KD), MAD, and low glycemic index treatment (LGIT) [47].

A ketogenic diet induces a metabolic state of ketosis through high fat, adequate protein, and very low carbohydrate intake. Traditionally, the therapeutic effects of KDTs were mainly attributed to ketone body production and altered cerebral energy metabolism. However, growing evidence suggests that ketogenic therapies may exert broader neuroprotective effects involving mitochondrial function, oxidative stress reduction, neurotransmitter modulation, neuroinflammation, and gut microbiota remodeling. In this context, ketogenic therapies are increasingly being reconsidered not only as metabolic interventions, but also as modulators of the microbiota–gut–brain axis.

#### 5.3.2. Gut Microbiota Remodeling During Ketogenic Therapies

Diet profoundly influences gut microbiota composition and activity, and KDTs induce distinct microbial and metabolic changes that may contribute to seizure control. Several studies have shown that ketogenic therapies reduce carbohydrate-degrading bacteria and overall microbial diversity while enriching taxa such as *Bacteroides*, *Prevotella*, and *Akkermansia*, alongside reductions in *Escherichia coli*. These microbial shifts are associated with altered production of microbial metabolites, including short-chain fatty acids (SCFAs), which are implicated in gut–brain axis modulation, neuroinflammation, and neuronal excitability.

Human studies further support these findings. Xie et al. [48] reported that one week of KD in children with refractory epilepsy increased *Bacteroides*, *Bifidobacterium*, and *Prevotella*, while decreasing *Cronobacter* and *Proteobacteria*, suggesting enhanced anti-inflammatory potential. Zhang et al. [49] observed that six months of KD induced microbial shifts in responders, including enrichment of *Alistipes*, *Lachnospiraceae*, *Ruminococcaceae*, and *Rikenellaceae*, associated with ≥50% seizure reduction.

Similarly, Gong et al. [50] reported increased fecal SCFA concentrations after six months of KD, together with enrichment of beneficial taxa and reduction in potentially pro-convulsive genera. At first glance, increased SCFA production during ketogenic dietary therapies may appear counterintuitive given the marked reduction in fermentable carbohydrate intake. However, this finding has not been reported consistently across studies and may depend on several factors, including the specific composition of the ketogenic diet, the residual intake of dietary fiber, and selective enrichment of microbial taxa with enhanced fermentative capacity. Consequently, changes in SCFA production should be interpreted cautiously, as they are likely influenced by both dietary composition and microbiota remodeling rather than carbohydrate restriction alone. Lindefeldt et al. further showed that KD significantly altered both microbial composition and metabolic pathways, particularly those involved in carbohydrate metabolism, indicating substantial microbial metabolic reprogramming [14].

Collectively, these findings suggest that ketogenic therapies induce profound remodeling of the intestinal ecosystem, potentially influencing seizure susceptibility through microbiota-derived metabolites, immune signaling, and modulation of neuroactive pathways [51].

#### 5.3.3. Microbiota-Mediated Antiseizure Mechanisms

Preclinical studies provide compelling evidence supporting microbiota-mediated antiseizure effects of ketogenic therapies. Olson et al. demonstrated that germ-free or antibiotic-treated mice failed to achieve seizure protection under KD, whereas fecal microbiota transplantation from KD-fed donors restored antiseizure effects. Moreover, co-colonization with *Akkermansia muciniphila* and *Parabacteroides* prevented seizures in germ-free mice, whereas single-species colonization did not, highlighting the importance of specific microbial interactions in mediating seizure protection.

Mechanistically, ketogenic therapies appear capable of modulating neurotransmitter balance, neuroinflammation, and blood–brain barrier integrity through microbial metabolites and gut-derived signaling pathways. Olson et al. also demonstrated that KD increased the hippocampal GABA/glutamate ratio, suggesting that microbiota-derived metabolites may directly influence inhibitory and excitatory neurotransmission [52].

Microbiota-derived SCFAs and other metabolites may further contribute to seizure protection by modulating neuronal excitability, reducing neuroinflammation, and promoting blood–brain barrier integrity. In addition, dietary fiber inclusion within ketogenic formulations may support SCFA-producing taxa, potentially enhancing the neuroprotective effects of ketogenic therapies.

#### 5.3.4. Clinical and Translational Perspectives

Despite promising clinical outcomes, the microbiota shifts induced by ketogenic therapies raise important questions regarding long-term intestinal homeostasis. Lindefeldt et al. observed reductions in health-promoting fiber-degrading bacteria during KD, suggesting potential trade-offs between antiseizure efficacy and gut ecosystem stability [14]. These observations also highlight a potential paradox of ketogenic dietary therapies. Although ketogenic diets may promote microbial changes associated with seizure control, they may simultaneously reduce overall microbial diversity and deplete taxa involved in fiber fermentation and maintenance of intestinal homeostasis. The long-term clinical significance of these changes remains uncertain, as it is currently unclear whether they represent transient adaptations to the dietary intervention or potentially unfavorable alterations in the gut ecosystem. These considerations underscore the importance of optimizing ketogenic dietary protocols by preserving dietary quality and, where feasible, incorporating fiber-rich, microbiota-supportive foods without compromising therapeutic efficacy.

These findings suggest that persistent microbial alterations induced by prolonged ketogenic therapies could become maladaptive over time, particularly if dietary approaches rely heavily on processed ketogenic products or low-fiber formulations that may exacerbate intestinal inflammation. Conversely, the use of fresh, high-quality whole foods and fiber-enriched ketogenic strategies may better preserve microbial diversity and intestinal homeostasis.

Overall, growing evidence supports the concept that ketogenic therapies act beyond ketosis alone, inducing complex metabolic, microbial, and neuroimmune remodeling. Future research should aim to identify microbiota signatures associated with treatment response while also clarifying the long-term consequences of ketogenic diet-induced microbial remodeling, to optimize dietary protocols that preserve both antiseizure efficacy and intestinal ecosystem integrity [50,51].

### 5.4. Mediterranean Diet and Fiber-Rich Dietary Patterns

#### 5.4.1. Microbiota-Supportive and Anti-Inflammatory Effects

Since the landmark Seven Countries Study over 50 years ago, the Mediterranean diet has been recognized as a model of preventive nutrition and health promotion [53]. This dietary pattern emphasizes unprocessed plant-based foods, olive oil, legumes, whole grains, fruits, vegetables, fish, and moderate dairy intake, while limiting red meat and ultra-processed foods.

Several studies have demonstrated that adherence to the Mediterranean diet is associated with enrichment of beneficial microbial taxa, including *Faecalibacterium prausnitzii* and *Roseburia* spp., together with reduction in potentially pro-inflammatory bacteria such as *Ruminococcus gnavus* and *Collinsella aerofaciens* [53,54,55]. These microbial shifts are associated with increased production of short-chain fatty acids (SCFAs), particularly butyrate, which contribute to intestinal barrier integrity, immune regulation, and anti-inflammatory signaling.

SCFAs, including acetate, propionate, and butyrate, exert pleiotropic effects through G protein-coupled receptors and modulation of enteroendocrine signaling. In particular, butyrate supports colonocyte metabolism, enhances mucus production, reinforces epithelial barrier integrity, and reduces inflammatory activation, while lower intestinal pH limits the growth of pathogenic taxa such as *Clostridia* and *Enterobacteriaceae* [56]. However, unlike KD, evidence supporting Mediterranean dietary patterns in epilepsy is currently indirect and largely derived from studies evaluating metabolic health, inflammation, and microbiota composition rather than syndrome-specific epilepsy outcomes.

#### 5.4.2. Potential Implications in Epilepsy

Compared with ketogenic dietary therapies, evidence directly evaluating the Mediterranean diet in epilepsy remains limited. However, growing interest surrounds its potential role as a microbiota-supportive and anti-inflammatory dietary model capable of modulating the microbiota–gut–brain axis.

Kaner et al. evaluated adherence to the Mediterranean diet in 85 children with epilepsy and observed positive associations between Mediterranean dietary patterns and intake of fiber, minerals, and micronutrients relevant to metabolic and intestinal health [57]. Although direct effects on seizure control were not specifically demonstrated, these findings suggest that fiber-rich dietary patterns may contribute to improved intestinal homeostasis and metabolic balance in pediatric epilepsy.

Overall, based on the currently available indirect evidence, the Mediterranean diet may represent a promising complementary dietary approach aimed at supporting gut microbial diversity, reducing neuroinflammatory signaling, and promoting long-term metabolic health in patients with epilepsy. Its high fiber content and ability to promote short-chain fatty acid (SCFA)-producing taxa may contribute to improved intestinal barrier integrity, immune regulation, and modulation of neuroinflammatory pathways increasingly implicated in epileptogenesis and epilepsy-related comorbidities [56]. Furthermore, Mediterranean dietary patterns have been associated with beneficial effects on cognitive and mental health outcomes, supporting their potential relevance as long-term microbiota-supportive interventions in neurological disorders [53,56].

Rather than acting as a direct antiseizure therapy, the Mediterranean diet may therefore represent a sustainable complementary nutritional strategy capable of modulating metabolic, inflammatory, and microbiota-related pathways involved in the microbiota–gut–brain axis. However, further clinical and mechanistic studies are needed to clarify its potential role in seizure modulation and treatment responsiveness.

Future personalized nutritional approaches in epilepsy may therefore integrate microbiota-supportive dietary models, ketogenic strategies, fiber supplementation, and metabolite-targeted interventions according to individual inflammatory and microbial profiles.

Although direct antiseizure evidence remains limited, the Mediterranean diet may represent a useful long-term microbiota-supportive framework capable of complementing ketogenic interventions once seizure stability is achieved.

### 5.5. Western Dietary Patterns, Dysbiosis, and Neuroinflammatory Signaling

The Western diet, characterized by high caloric intake, elevated levels of animal protein, saturated fats, simple sugars, and ultra-processed foods, combined with low consumption of fiber, fruits, and vegetables, exerts profound effects on gut microbiota. This dietary pattern is associated with reduced microbial diversity and altered microbial configurations frequently associated with pro-inflammatory metabolic profiles and impaired intestinal homeostasis. Additional taxa commonly enriched include *Ruminococcus*, *Faecalibacterium*, *Bifidobacterium*, *Alistipes*, *Blautia*, and *Bilophila*. At the same time, depletion of fiber intake and disruption of SCFA-producing microbial communities may contribute to reduced production of beneficial short-chain fatty acids (SCFAs), particularly butyrate, with potential consequences for intestinal barrier integrity, immune regulation, and neuroinflammatory signaling.

Compounds abundant in red meat, such as choline and carnitine, can be metabolized by gut microbes into trimethylamine (TMA), which is converted in the liver to trimethylamine N-oxide (TMAO), a metabolite linked to chronic diseases [58,59]. Beyond macronutrient composition alone, ultra-processed foods characteristic of Western dietary patterns contain additives, preservatives, emulsifiers, and artificial sweeteners capable of directly or indirectly modulating microbial ecology and intestinal inflammatory pathways [60]. Non-nutritive sweeteners, including saccharin, sucralose, and aspartame, may alter microbial diversity over time, though their long-term impact remains unclear. Other additives, such as carrageenan, can disrupt the intestinal mucus layer and promote gut inflammation, while artificial colorants, like Allura Red AC, can influence microbial sulfur metabolism. Preservatives (e.g., sodium nitrate) and emulsifiers (e.g., carboxymethylcellulose, polysorbate-80) also impact microbial composition and function [61].

Overall, the Western diet promotes chronic low-grade inflammation, intestinal barrier dysfunction, and maladaptive MGBA signaling, and is associated with increased risk of obesity and a broad spectrum of non-communicable diseases. Emerging evidence suggests that Western dietary patterns may contribute to neuroinflammatory activation and altered neuronal excitability through microbiota-mediated immune and metabolic pathways [61,62]. Reduced microbial resilience, depletion of SCFA-producing taxa, increased intestinal permeability, and systemic inflammatory activation may collectively favor conditions associated with increased neuronal vulnerability and epileptogenesis. By promoting systemic inflammation, microglial activation, and disruption of blood–brain barrier integrity, Western dietary patterns may lower the seizure threshold and facilitate neuronal hyperexcitability, providing a plausible mechanistic link between diet-induced dysbiosis and epileptogenesis [6,63].

In contrast to microbiota-supportive dietary models such as the Mediterranean diet, long-term adherence to Western dietary patterns may therefore impair intestinal ecosystem stability and metabolic flexibility, potentially exacerbating inflammatory and neuroimmune pathways implicated in epilepsy and its comorbidities. From an epilepsy perspective, these observations are particularly relevant because chronic systemic inflammation, impaired intestinal barrier function, and altered microbial metabolite production have all been implicated in lowering the seizure threshold and promoting neuroinflammatory pathways. Although direct clinical evidence linking Western dietary patterns to seizure frequency remains limited, the biological mechanisms associated with this dietary pattern provide a plausible rationale for considering dietary quality as a potential modifiable factor in epilepsy management.

Although direct evidence linking Western dietary patterns to seizure susceptibility and clinical outcomes in epilepsy remains limited, the available mechanistic evidence supports further investigation of dietary quality as a potential modifiable factor influencing neuroinflammation and seizure risk.

### 5.6. Restrictive Diets in Epilepsy

Currently, there is limited and inconsistent evidence supporting the use of restrictive dietary protocols, such as gluten-free or lactose-free diets, for patients with epilepsy, unless there is a clearly documented clinical indication, such as celiac disease, lactose intolerance, or other verified food allergies. At present, empirical adoption of restrictive dietary regimens in epilepsy has not been consistently associated with improved seizure control, gut microbiota diversity, or neurological outcomes in patients without specific medical indications. On the contrary, unnecessary dietary restrictions may increase the risk of malnutrition due to micronutrient deficiencies, while also reducing dietary diversity and potentially impairing microbiota-supportive nutrient intake. These alterations may negatively affect intestinal ecosystem stability, metabolic homeostasis, and overall health status.

Gluten-free diets, recommended only for patients with celiac disease according to ESPGHAN guidelines, often lead to higher consumption of processed foods and may result in deficiencies of folate (B9), thiamine (B1), niacin (B3), and vitamin B12, as many industrial gluten-free products are made with unfortified refined flours [63,64,65]. Similarly, prolonged lactose-free regimens may compromise calcium and vitamin D intake when not appropriately supervised, while empiric casein exclusion lacks sufficient evidence in epilepsy outside specific clinical indications [66,67]. Furthermore, most commercially available gluten-free products targeted at children are high in sugars, fat, and sodium, with up to 98% exceeding nutrient thresholds set by PAHO (Pan American Health Organization) [68]. Importantly, reliance on fortified ultra-processed “free-from” products to compensate for nutritional deficiencies may paradoxically increase exposure to additives, refined sugars, emulsifiers, and industrial vegetable oils, all of which may further influence gut microbial composition and inflammatory pathways [69]. From a microbiota-supportive perspective, a preferable strategy may consist of emphasizing naturally nutrient-dense and minimally processed foods such as pseudocereals, eggs, dairy products, fish, poultry, and organ meats. These foods provide essential vitamins and minerals while avoiding unnecessary dietary restrictions.

However, it should be acknowledged that most of the available evidence regarding restrictive diets derives from the broader fields of nutrition and gastroenterology rather than epilepsy-specific studies. Consequently, although these findings provide useful insights into the potential nutritional and microbiota-related consequences of unnecessary dietary restrictions, their direct implications for seizure control and epilepsy outcomes remain insufficiently established.

Overall, current epilepsy-specific evidence does not support the routine implementation of restrictive dietary regimens in the absence of clearly documented medical indications. While general nutritional evidence highlights the potential risks of unnecessary dietary restrictions, further epilepsy-focused studies are needed to determine their impact on seizure control, gut microbiota, and long-term neurological outcomes.

**Figure 1:**
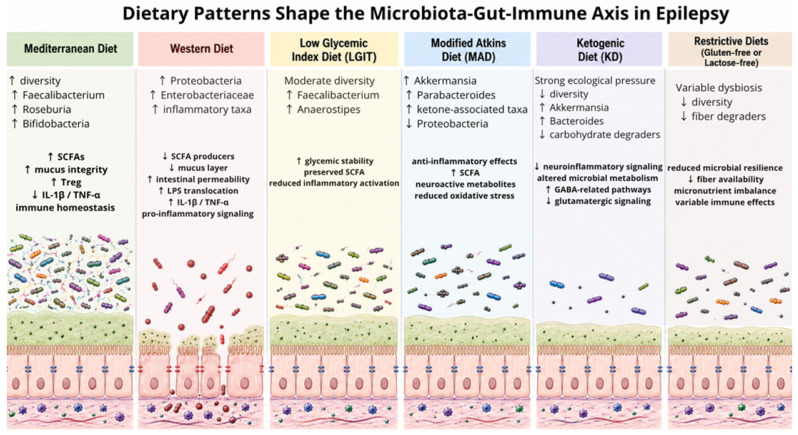
Effects of different dietary patterns on gut microbiota composition, intestinal barrier integrity, microbial metabolites, and immune responses involved in the microbiota–gut–immune axis in epilepsy. ↑ Increase; ↓ decrease.

**Figure 2:**
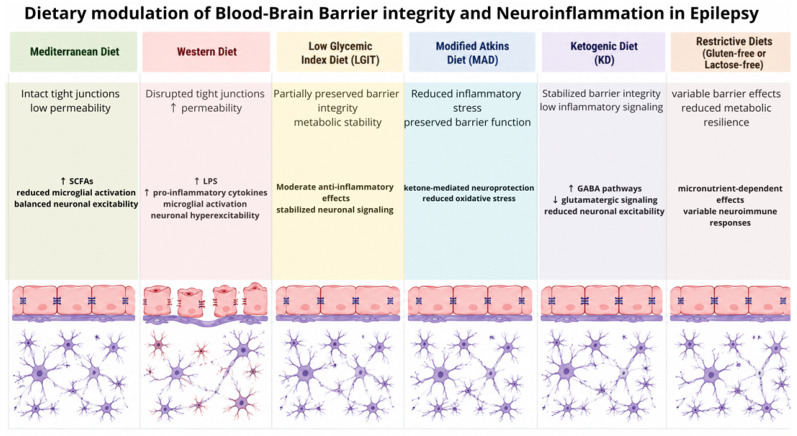
Impact of different dietary patterns on blood–brain barrier integrity, neuroinflammation, and neuronal excitability in epilepsy. ↑ Increase; ↓ decrease.

While the MGBA represents the overarching framework of bidirectional communication between the gut and the central nervous system, immune signaling constitutes one of its principal mechanistic components. Therefore, the microbiota–gut–immune axis should be viewed as an interconnected pathway within the broader MGBA rather than as a distinct biological entity. Through modulation of intestinal barrier integrity, systemic inflammation, cytokine signaling, and neuroimmune responses, immune mechanisms provide a critical interface linking gut microbial activity to brain function and seizure susceptibility. For clarity, the proposed effects of different dietary patterns on the microbiota–gut–immune axis, blood–brain barrier integrity, and neuroinflammatory pathways relevant to epilepsy are schematically illustrated in Figure 1 and Figure 2. The figures are intended to summarize the current mechanistic hypotheses derived from available experimental and clinical evidence and should not be interpreted as representing fully established causal pathways.

## 6. Integrating Microbiota-Targeted Supplementation into Epilepsy Management

### 6.1. Probiotics, Prebiotics, and Postbiotics: Biological Functions Within the Microbiota–Gut–Brain Axis

Microbiota-targeted supplementation has emerged as a promising complementary strategy within the microbiota–gut–brain axis (MGBA) framework, aiming to modulate intestinal homeostasis, microbial metabolite production, neuroimmune signaling, and host physiology. Probiotics are defined by the World Health Organization (WHO) as live microorganisms that, when administered in adequate amounts, confer health benefits to the host. These microbes influence the host through multiple mechanisms, including modulation of immune responses and maintenance of a balanced intestinal environment. By shaping gut microbiota composition, probiotics can promote the persistence of beneficial microbial populations [70]. Similarly, prebiotics are substrates selectively utilized by host microorganisms to confer health benefits [71]. These include non-digestible oligosaccharides, soluble fermentable fibers, and human milk oligosaccharides (HMOs). Since human digestive enzymes cannot degrade these compounds, they reach the colon largely intact, where commensal bacteria ferment them into SCFA such as acetate, propionate, and butyrate. SCFAs represent one of the best-characterized classes of postbiotic metabolites generated through microbial fermentation and capable of modulating intestinal barrier integrity, immune homeostasis, and neuroinflammatory signaling [72]. Collectively, probiotics, prebiotics, and postbiotics interact dynamically in regulating the MGBA and may influence inflammatory, metabolic, and neuroimmune pathways increasingly implicated in epilepsy and neurological disorders.

### 6.2. Neurotransmitter Modulation and Gut–Brain Communication

Probiotics, prebiotics, and postbiotics are closely interconnected in their modulation of the MGBA. Certain probiotic strains, sometimes referred to as “psychobiotics,” may directly or indirectly modulate neuroactive pathways through production of neurotransmitters and microbial metabolites capable of influencing gut–brain communication. For example, GABA is secreted by specific *Lactobacillus* and *Bifidobacterium* strains, norepinephrine by *Saccharomyces*, *Bacillus*, and *Escherichia*, serotonin by *Enterococcus*, *Streptococcus*, *Escherichia*, and *Candida*, and dopamine by *Bacillus* and *Serratia* [71,72]. Several experimental studies in rodents have demonstrated that probiotics can modulate brain neurochemistry and behavior. For instance, administration of *Lactobacillus rhamnosus* (JB-1) increased GABA and glutamate levels in the brain, altered GABA receptor expression, and regulated stress-induced behaviors [73]. Similarly, *Bifidobacterium infantis* normalized immune responses, restored basal noradrenaline concentrations, and modulated dopamine and serotonin metabolites in the frontal cortex of rats [74]. Collectively, these findings support the concept that gut microorganisms may influence neuronal signaling, stress responsiveness, and neuroimmune homeostasis through bidirectional pathways involving microbial metabolites, neurotransmitters, vagal signaling, and immune mediators within the microbiota–gut–brain axis.

Nevertheless, the current evidence base is still constrained by the paucity of adequately powered randomized controlled trials, preventing definitive conclusions regarding the clinical efficacy of microbiota-targeted supplementation in epilepsy.

### 6.3. SCFAs, Epigenetic Regulation, and Neuroinflammation

SCFAs mediate diverse effects on host physiology. They regulate histone acetylation and methylation [75], stimulate enteroendocrine secretion of hormones such as Glucagon-Like Peptide-1 (GLP-1) and peptide YY (PYY), and modulate neurotransmitter levels, including serotonin [76]. SCFAs also activate vagus nerve signaling [77] and reinforce intestinal barrier integrity, preventing bacterial translocation [78]. Butyrate, in particular, serves as an energy source for colonocytes, enhances mucus production, modulates immune responses via regulatory T cells, and influences systemic energy homeostasis, obesity, cancer, inflammation, and brain function [79]. Furthermore, butyrate contributes to gut–brain communication through interactions involving the enteric nervous system, vagus nerve signaling, and the hypothalamic–pituitary–adrenal axis [80]. At pharmacological concentrations, butyrate exerts systemic anti-inflammatory and epigenetic effects through mechanisms including histone deacetylase (HDAC) inhibition and NF-κB modulation [81]. Although direct brain exposure to butyrate derived from dietary fermentation remains limited, enhanced SCFA production induced by diet, probiotics, or prebiotic supplementation consistently supports intestinal barrier integrity, immune regulation, and systemic metabolic homeostasis. Emerging evidence suggests that these mechanisms may also contribute to the modulation of neuroinflammatory pathways and neuronal excitability within the MGBA (Table 1).

### 6.4. Translational Perspectives in Epilepsy

Collectively, growing evidence suggests that microbiota-targeted supplementation may influence neuroinflammation, intestinal permeability, and microbiota–gut–brain axis signaling. However, current evidence in epilepsy remains preliminary and is derived predominantly from preclinical studies, with only limited clinical data available. Consequently, although probiotics, prebiotics, and postbiotics represent promising investigational approaches, their routine clinical use in epilepsy cannot currently be recommended, and further well-designed clinical trials are needed to establish their efficacy and safety.

Future personalized therapeutic approaches may integrate microbiota-targeted supplementation with ketogenic therapies, fiber-enriched nutritional strategies, and metabolite-based interventions according to individual microbial, metabolic, and inflammatory profiles. Further clinical and mechanistic studies are needed to clarify strain-specific effects, optimal therapeutic combinations, long-term safety, and their potential impact on seizure control and epilepsy-related comorbidities.

## 7. Future Directions and Precision Nutrition in Epilepsy

Future research is expected to move beyond standardized dietary interventions toward precision nutrition approaches tailored to individual biological characteristics. As the gut microbiota is increasingly recognized as a central regulator of host metabolism, immunity, and brain function, understanding its complex networks may provide an unprecedented opportunity to modulate human physiology through targeted nutritional interventions. In this context, deciphering the microbiome may represent a form of biological decoding of the human host, thus enabling a deeper understanding of the mechanisms that link diet, microbial ecosystems, and neuronal function [8].

Advances in multi-omics technologies, including metagenomics, transcriptomics, and metabolomics, together with artificial intelligence and machine learning approaches, may enable the integration of microbial, metabolic, and clinical data into comprehensive digital representations of individual microbiome ecosystems [84]. These microbiome-informed models could facilitate the identification of predictive biomarkers, improve patient stratification, and support the development of personalized dietary interventions [85].

Ultimately, the convergence of omics sciences, computational biology, and precision nutrition may allow clinicians to target specific microbial and metabolic pathways, transforming dietary therapy from a generalized intervention into a mechanism-based and individualized component of epilepsy management.

## 8. Current Evidence Gaps and Methodological Challenges

Despite growing interest in the MGBA as a potential therapeutic target in epilepsy, current evidence remains limited by several methodological challenges. Many available studies are characterized by small sample sizes, heterogeneous patient populations, differences in epilepsy etiology, age, antiseizure medications, and dietary interventions, limiting comparability across studies. Furthermore, substantial heterogeneity exists regarding epilepsy syndromes, disease etiologies, and treatment responsiveness, making it difficult to determine whether microbiota-related alterations are shared across epilepsy subtypes or are specific to particular clinical phenotypes.

An additional source of heterogeneity relates to the age distribution of the study populations. Many microbiota and dietary intervention studies have been conducted predominantly in pediatric cohorts, whereas adult data remain comparatively limited. Consequently, extrapolation of the findings across age groups should be interpreted with caution, and future studies should further investigate age-specific microbiota signatures and therapeutic responses.

Moreover, dietary habits, lifestyle factors, antibiotic exposure, and other environmental influences represent important confounding variables that may substantially affect gut microbiota composition and metabolic profiles. Variability in microbiome-sequencing methods, bioinformatic analyses, and outcome measures further contributes to inconsistencies across studies.

Most importantly, the majority of available evidence remains observational, making it difficult to establish causal relationships between gut microbial alterations, dietary interventions, and seizure control. Future large-scale, longitudinal, and multi-omics studies are needed to better define the mechanistic pathways linking the microbiota–gut–brain axis to epilepsy and to identify reliable biomarkers for personalized therapeutic approaches. An important unresolved issue concerns the lack of a reproducible microbial signature capable of consistently distinguishing treatment responders from non-responders across studies. Although several investigations have identified associations between specific microbial taxa and clinical outcomes, these findings have rarely been replicated in independent cohorts. This variability likely reflects differences in patient populations, epilepsy syndromes, antiseizure medications, dietary interventions, sequencing methodologies, and analytical approaches. Furthermore, treatment response may depend less on the presence or absence of individual microbial taxa than on broader functional and metabolic characteristics of the gut microbiome. Future studies integrating metagenomic, metabolomic, and longitudinal analyses will be essential to identify robust microbiome-based biomarkers of treatment response.

## 9. Conclusions

Growing evidence on the MGBA is progressively reshaping the understanding of dietary therapies in epilepsy. These interventions should no longer be viewed exclusively as metabolic strategies aimed at inducing ketosis but may also influence gut microbial ecology, neuroimmune signaling, and intestinal homeostasis. In this context, ketogenic and microbiota-supportive dietary approaches may influence seizure susceptibility through interconnected metabolic, immune, and microbial mechanisms. Nevertheless, much of the current evidence remains associative, and the precise contribution of microbiota-mediated mechanisms to the clinical efficacy of dietary therapies has yet to be fully established. Further well-designed longitudinal and mechanistic studies will be essential to translate these promising findings into clinical practice.

Future research should focus on integrative clinical studies combining dietary interventions with microbiome analysis, metabolomics, and other multi-omics approaches to identify microbial and inflammatory signatures associated with treatment response and to develop more personalized microbiota-targeted strategies.

Importantly, microbiota-directed interventions, including individualized nutrition and the use of pro-, pre-, and postbiotics, should not be considered as alternatives to pharmacological therapy, but rather as complementary strategies that should proceed in parallel with conventional antiseizure treatments. Dietary therapy should therefore be reconsidered not as a “last-resort” option introduced only after pharmacological and clinical failure, but as a valuable adjunctive strategy that may complement conventional antiseizure treatments within a personalized medicine framework.

## Figures and Tables

**Table 1:** Proposed mechanisms and experimental evidence supporting microbiota-targeted interventions in epilepsy and gut–brain communication.

Intervention	Main Mechanism(s)	Key Findings	References
**Probiotics (*Lactobacillus* and *Bifidobacterium* spp.)**	Modulation of gut microbiota composition, immune regulation, production of neuroactive compounds	Promotion of beneficial microbial populations and maintenance of intestinal homeostasis within the MGBA	[68]
**Prebiotics (fermentable fibers, oligosaccharides, HMOs)**	Selective stimulation of beneficial bacteria and SCFA production	Increased microbial fermentation leading to acetate, propionate, and butyrate production, with potential effects on barrier integrity and immune regulation	[69,70]
**Postbiotics (SCFAs, particularly butyrate)**	Epigenetic regulation (HDAC inhibition), immune modulation, intestinal barrier support	Modulation of neuroinflammatory pathways, enhancement in gut barrier function, and regulation of host metabolism	[73,74,75,76,77,78,79]
***Lactobacillus rhamnosus* (JB-1)**	Gut–brain signaling through neurotransmitter modulation	Increased brain GABA and glutamate levels, altered GABA receptor expression, and reduced stress-related behaviors in animal models	[82]
***Bifidobacterium infantis***	Neuroimmune and neurotransmitter modulation	Restoration of basal noradrenaline levels and modulation of dopamine and serotonin metabolites in the frontal cortex of rats	[83]
**Psychobiotic strains (*Lactobacillus*, *Bifidobacterium*, *Saccharomyces*, *Bacillus*, *Enterococcus* spp.)**	Production of neuroactive molecules including GABA, serotonin, dopamine, and norepinephrine	Potential modulation of neuronal signaling and gut–brain communication pathways	[71,72]
**SCFA-producing microbiota induced by probiotics/prebiotics**	Vagal signaling, enteroendocrine activation (GLP-1, PYY), anti-inflammatory effects	Reinforcement of intestinal barrier integrity and modulation of neuroimmune pathways potentially relevant to seizure susceptibility	[74,75,76,77,78]

## Data Availability

No new data were created or analyzed in this study.

## Abbreviations

The following abbreviations are used in this manuscript:

ASM	Antiseizure Medication
ASD	Autism Spectrum Disorder
DRE	Drug-Resistant Epilepsy
GABA	Gamma-Aminobutyric Acid
GLP-1	Glucagon-Like Peptide-1
HDAC	Histone Deacetylase
HMO	Human Milk Oligosaccharide
KD	Ketogenic Diet
KDTs	Ketogenic Dietary Therapies
KATP	ATP-Sensitive Potassium Channel
LGIT	Low Glycemic Index Treatment
LPS	Lipopolysaccharide
MAD	Modified Atkins Diet
MCT-KD	Medium-Chain Triglyceride Ketogenic Diet
MGBA	Microbiota–Gut–Brain Axis
NF-κB	Nuclear Factor Kappa B
PNESs	Psychogenic Non-Epileptic Seizures
PYY	Peptide YY
SCFAs	Short-Chain Fatty Acids
WHO	World Health Organization
